# Systemic Administration of Tempol Attenuates the Cardiorespiratory Depressant Effects of Fentanyl

**DOI:** 10.3389/fphar.2021.690407

**Published:** 2021-06-23

**Authors:** Santhosh Baby, Ryan Gruber, Joseph Discala, Veljko Puskovic, Nijo Jose, Feixiong Cheng, Michael Jenkins, James Seckler, Stephen Lewis

**Affiliations:** ^1^ Galleon Pharmaceuticals, Inc., Horsham, PA, United States; ^2^ Department of Radiotherapy and Oncology, Kasturba Medical College, Manipal, India; ^3^ Genomic Medicine Institute, Lerner Research Institute, Cleveland Clinic, Cleveland, OH, United States; ^4^ Department of Biomedical Engineering, Case Western Reserve University, Cleveland, OH, United States; ^5^ Department of Pediatrics, Case Western Reserve University, Cleveland, OH, United States

**Keywords:** tempol, fentanyl, cardiorespiratory depression, superoxide dismutase, sprague-dawley rats

## Abstract

Fentanyl is a high-potency opioid receptor agonist that elicits profound analgesia and suppression of breathing in humans and animals. To date, there is limited evidence as to whether changes in oxidant stress are important factors in any of the actions of acutely administered fentanyl. This study determined whether the clinically approved superoxide dismutase mimetic, Tempol (4-hydroxy-2,2,6,6-tetramethylpiperidine-N-oxyl), or a potent antioxidant, N-acetyl-L-cysteine methyl ester (L-NACme), modify the cardiorespiratory and analgesic actions of fentanyl. We examined whether the prior systemic injection of Tempol or L-NACme affects the cardiorespiratory and/or analgesic responses elicited by the subsequent injection of fentanyl in isoflurane-anesthetized and/or freely moving male Sprague-Dawley rats. Bolus injections of Tempol (25, 50 or 100 mg/kg, IV) elicited minor increases in frequency of breathing, tidal volume and minute ventilation. The ventilatory-depressant effects of fentanyl (5 μg/kg, IV) given 15 min later were dose-dependently inhibited by prior injections of Tempol. Tempol elicited dose-dependent and transient hypotension that had (except for the highest dose) resolved when fentanyl was injected. The hypotensive responses elicited by fentanyl were markedly blunted after Tempol pretreatment. The analgesic actions of fentanyl (25 μg/kg, IV) were not affected by Tempol (100 mg/kg, IV). L-NACme did not modify any of the effects of fentanyl. We conclude that prior administration of Tempol attenuates the cardiorespiratory actions of fentanyl without affecting the analgesic effects of this potent opioid. As such, Tempol may not directly affect opioid-receptors that elicit the effects of fentanyl. Whether, the effects of Tempol are solely due to alterations in oxidative stress is in doubt since the powerful antioxidant, L-NACme, did not affect fentanyl-induced suppression of breathing.

## Introduction

Fentanyl is high-potency opioid receptor (OR) agonist that is widely used to treat both acute and chronic pain (Suzuki and El-Haddad, 2017; Armenian et al., 2018). The misuse/abuse of fentanyl and analogues such as sufentanil and carfentanil leads to adverse consequences, including often lethal depression of ventilation (Suzuki and El-Haddad, 2017; Armenian et al., 2018). Fentanyl is classified as a selective μ-OR agonist and has high affinity for μ-ORs (Raynor et al., 1994; Lipiński et al., 2019). However, fentanyl also activates δ- and κ-ORs with affinities and intrinsic activities of biological significance (Yeadon and Kitchen, 1990; Zhu et al., 1996; Gharagozlou et al., 2006). For example, whereas fentanyl has low affinity for κ-ORs it has a remarkably high efficacy at these receptors (Gharagozlou et al., 2006). The mechanisms responsible for the ventilatory depressant and analgesic effects of fentanyl and analogues have been studied extensively (Mayer et al., 1989; Dahan et al., 2010). Henderson et al. (2014) reported that pre-treatment of rats with naloxone methiodide, a peripherally restricted μ-OR antagonist, attenuated fentanyl-induced analgesia, decreases in tidal volume (TV) and increases in Alveolar-arterial (A-a) gradient (indicative of ventilation-perfusion mismatch/shunting in the lungs). As such, it is likely that the pharmacological actions of fentanyl involve a mixture of effects in the periphery (e.g., vagal cardiopulmonary afferents, the chest-wall and carotid bodies), brain regions such as the area postrema that are devoid of a blood-brain barrier, and also brain structures within the blood brain barrier such as the nucleus tractus solitarius (Mayer et al., 1989; Dahan et al., 2010; Henderson et al., 2014).

There is conflicting evidence as to whether opioids induce oxidative stress. For example, there is compelling evidence that morphine, buprenorphine and methadone can both increase or decrease oxidative stress depending on the circumstances and experimental conditions (Lee et al., 2004; Almeida et al., 2014; Motaghinejad et al., 2015; Skrabalova et al., 2018; Leventelis et al., 2019). In contrast, the available evidence suggested that fentanyl and analogues either reduce (Kim et al., 2017) or have no effect (Krumholz et al., 1993; Jaeger et al., 1998) on oxidative stress although there is a report that a high-dose remifentanil increases myocardial oxidative stress and compromises remifentanil infarct-sparing effects in rats (Mei et al., 2013). In order, to address the importance of superoxide anion in the pharmacological actions of fentanyl, we determined the effects of pretreating rats with the stable cell permeable superoxide dismutase-mimetic and free radical scavenger, Tempol (4-hydroxy-2,2,6,6-tetramethylpiperidine-N-oxyl) (Wilcox and Pearlman, 2008; Wilcox, 2010; Kim et al., 2016), on the cardiorespiratory and analgesic effects elicited by injections of fentanyl. Previous studies have demonstrated beneficial effects of Tempol in cell and animal models of tumororigenesis, shock, hypertension, diabetes, ischemia-reperfusion injury, traumatic brain injury, neurodegenerative diseases, chemotherapy-induced neuropathic pain, and alopecia (Wilcox and Pearlman, 2008; Wilcox, 2010; Kim et al., 2016; Bernardy et al., 2017; Wang et al., 2018; Chiarotto et al., 2019; Afjal et al., 2019). To support these studies, we have examined whether the cell permeable antioxidant, N-acetyl-L-cysteine methyl ester (L-NACme) (Michaelsen et al., 2009; Giustarini et al., 2012; Poopari et al., 2015; Giustarini et al., 2018; Uemura et al., 2018) modulated the ventilatory depressant actions of fentanyl (see Supplementary Figure S1 for depiction of Tempol, L-NAC and L-NACme structures). The doses of Tempol were chosen on the basis of *in vivo* studies performed by other groups and especially those done in rats (i.e., Xu et al., 2004; Xu et al., 2006; Wilcox and Pearlman, 2008; Kim et al., 2016).

By definition, effective OR antagonists will block/reverse all of the pharmacological actions of opioids including the necessary/desired analgesic effects. At present, drugs that can effectively reverse the negative effects of opioids on breathing and arterial blood pressure without affecting opioid-induced analgesia are lacking (see Dahan et al., 2018). As such, this clinical gap may be met by drugs with the pharmacological profile of Tempol and the yet untested array of available Tempol analogues.

## Materials and Methods

### Permissions

All animal studies were carried out in accordance with the National Institutes of Health Guide for the Care and Use of Laboratory Animals (NIH Publication No. 80.23) revised in 1996. The protocols were approved by the Institutional Animal Care and Use Committee at Galleon Pharmaceuticals, Inc. (Horsham, PA, United States), and Case Western Reserve University (Cleveland, OH, United States). Adult male Sprague-Dawley rats (300–350 g) from Harlan Laboratories, Inc. (Indianapolis, IN, United States) were used for this study. The rats were caged in a Innocage IVC rat caging system (InnoVive, San Diego, CA, United States) in our vivariums in rooms with a 12 h light-dark cycle and standard housing conditions, namely, room temperature of 22°C and relative humidity of 35–40%. The rats had free access to water and standard rat chow.

### Drugs

Saline (vehicle) and fentanyl (50 μg/ml ampoules) were purchased from Hospira Inc. (Lake Forest, IL, United States). Tempol was purchased from Tocris (Minneapolis, MN, United States). L-NACme was purchased from Sigma-Aldrich (St. Louis, MO, United States).

### Cardiorespiratory Measurements in Anesthetized Rats

The effects of bolus intravenous (IV) injections of vehicle (saline), Tempol (25, 50 or 100 mg/kg; stock solutions of Tempol of 100 mg/ml in 0.9% saline were prepared freshly) and fentanyl (5 μg/kg) on heart rate (HR) and mean (MAP), diastolic (DBP) and systolic (SBP) blood pressures, and frequency of breathing (Freq), tidal volume (TV) and minute ventilation (MV) were evaluated in anesthetized spontaneously breathing rats by methods described previously (Dallas et al., 2015; Golder et al., 2015). In brief, rats were anesthetized with 2–2.5% isoflurane in compressed air. A length of PE-50 tubing (Instech Laboratories, Inc.) was inserted into a femoral vein to inject drugs. The venous line was connected to saline filled three-way connector for fluid support (4 ml/kg/h; 1:1 mixture of lactated Ringer’s and 6% Hetastarch) and for IV injection of Tempol and fentanyl. The cervical trachea was exposed ventrally, and a length of PE240 tubing (Instech Laboratories, Inc., Plymouth Meeting, PA) was inserted and sutured in place. The tracheal tube was connected to a pneumotachometer (MLT1L, AD instruments, Inc., CO) and a differential pressure transducer (FE141, AD instruments, Inc.) to measure respiratory flow via a T-shaped connector. The free end of the connector was attached to the source of isoflurane in compressed medical grade air. After the surgical procedures, the rats breathed spontaneously on 1.5% isoflurane during the study. Body temperature was kept at 37°C using a thermal blanket (Harvard Apparatus, Holliston, MA, United States). Respiratory flow was used to measure the Freq from the cyclic periods and integrated to measure TV. MV was calculated by multiplying Freq by TV.

A length of PE-50 tubing (Instech Laboratories, Inc.) was inserted into a femoral artery in order to continuously record cardiovascular parameters. The arterial catheter was connected to heparinized-saline filled pressure transducer (SP844-28; Memscap Inc., NC) and arterial pressure signals were amplified using the bridge amplifier (FE221, AD Instruments, Inc.). The arterial blood pressure waveform was used for measuring HR, DBP, SBP and MAP using the cyclic algorithms in the LabChart software. Respiratory flow and blood pressure waveforms were digitized (PowerLab, AD Instruments, Inc.) and continuously recorded using LabChart 7 Pro Software (AD Instruments, Inc.). After completion of all the surgical procedures, baseline cardiorespiratory parameters were determined and recorded for 10 min once stable values occurred. The average of this 10 min period was determined and all subsequent (i.e., pre- and post-injection) values were expressed as a percentage of this average.

### Ventilatory Recordings in Freely Moving Rats

Ventilatory parameters were continuously recorded in unrestrained freely moving rats via a whole-body 12-chamber plethysmography system (PLY 3223; BUXCO Inc., Wilmington, NC, United States) as described previously (Henderson et al., 2013; May et al., 2013a; May et al., 2013b; Henderson et al., 2014; Dallas et al., 2015; Baby et al., 2018). In brief, each rat was placed in an individual plexiglass chamber and the venous line was attached to a swivel assembly on the roof of the chamber to allow drug injections. The respiratory flow waveform was derived from a pneumotachometer in the chamber wall. Respiratory flow, chamber temperature, and humidity were measured continuously and used to calculate TV using the Epstein and Epstein algorithm (Henderson et al., 2013; May et al., 2013a; May et al., 2013b; Henderson et al., 2014; Dallas et al., 2015; Baby et al., 2018). The respiratory flow waveform cycle period was used to calculate Freq.

### Analgesia Assessment by Paw Withdrawal Assay

The acute antinociceptive effects of Tempol and fentanyl were assessed on paw-withdrawal (PW) latency using the Hargreaves’s test (Hargreaves et al., 1988). Briefly, paw withdrawal latency to a thermal stimulus was assessed using a radiant heat source (IITC, CA, United States) aimed at the planter surface of the left hind-paw. A cut-off latency of 20 s was set to avoid tissue damage. This method involved no restraint while positioning the thermal stimulus (instrument setting of 50–75% active intensity), sufficient enough to induce a latency of tail withdrawal of 40 s (baseline values) prior to the injection of any drugs.

### Protocols

#### Fentanyl-Induced Cardiorespiratory Depression in the Anesthetized Model

The effects of Tempol on fentanyl-induced cardiorespiratory parameters were determined in anesthetized spontaneously breathing rats. After completion of the surgical procedures, baseline cardiorespiratory parameters were determined and the rats then received slow bolus injections of vehicle or Tempol (25, 50 or 100 mg/kg, IV). After 20 min, all of the rats received a bolus injection of fentanyl (5 μg/kg) and cardiorespiratory variables recorded for a further 15 min.

#### Analgesia Testing in Conscious Rats

To determine the effects of Tempol on analgesia, baseline PW latencies were established and the rats then received injections of vehicle (*n* = 6) or 1, 10 or 100 mg/kg doses of Tempol (*n* = 6 rats per dose). PW latency was again tested 20, 40, 60, 90 and 120 min after injection of saline or Tempol. To determine the effects of Tempol on fentanyl-induced analgesia, baseline PW latencies were established at 10 AM and rats received an injection of fentanyl (25 μg/kg, IV). Five min later, the rats received injections of vehicle (1 ml/kg, *n* = 6), or Tempol (100 mg/kg, *n* = 6). PW latencies were tested 20, 40, 60, 90 and 120 min following injections of vehicle or Tempol. Fentanyl (25 μg/kg, IV) was given again to both groups at 4 PM and PW latencies were tested at 20, 40, 60, 90 and 120 min. To determine the effects of N-acetyl-L-cysteine methyl ester (L-NACme), on fentanyl-induced analgesia, baseline PW latencies were established at 10 AM and rats received an injection of fentanyl (25 μg/kg, IV). Five min later, the rats received injections of vehicle (1 ml/kg, *n* = 6, IV), or L-NACme (500 μmol/kg, IV, *n* = 6). PW latencies were tested 20, 40, 60, 90, and 120 min following the injections of vehicle or Tempol. An injection of fentanyl (25 μg/kg, IV) was given again to both groups of rats at 4 PM and PW latencies were tested after 20, 40, 60, 90 and 120 min.

#### Ventilatory Studies in Freely Moving Rats

The effects of pretreatment with L-NACme on fentanyl-induced respiratory depression were evaluated in freely moving rats. The rats were placed in the plethysmography chambers and after a period of acclimatization, they received an injection of Vehicle (saline, *n* = 9) or L-NACme (500 μmol/kg, IV; *n* = 9) and after 10 min all rats received a bolus injection of fentanyl (25 μg/kg, IV). Ventilatory parameters were recorded for a further 15 min.

#### Sample Sizes and Data Analyses

With respect to determining the sample sizes in the cardiorespiratory studies, our strategy was to use enough rats to provide 1) a comprehensive set of control data from the vehicle + fentanyl-injected rats (*n* = 12 rats), 2) enough rats to determine the trend with each dose (e.g., three rats for the 25 mg/kg dose of Tempol was sufficient to show a clear lack of effect against fentanyl), and 3) to use many more rats (*n* = 12) in the studies using the 100 mg/kg dose of Tempol that provided the most efficacy in order to allow for robust comparisons of the data to that from the vehicle-treated rats. In the analgesia studies, our approach was to keep using rats until the data clearly provided for valid statistical comparisons (i.e., in these studies, six rats per group). All data are presented as mean ± SEM and were evaluated using one-way and two-way ANOVA followed by Bonferroni corrections for multiple comparisons between means using the error mean square term from the ANOVA (Wallenstein et al., 1980). Differences between means were considered significant at *p* < 0.05. Statistical analyses were performed using GraphPad Prism software (GraphPad Software, Inc., La Jolla, CA, Unites States).

## Results

### Baseline Values and Derivation of %Changes in Cardiorespiratory Variables

**FIGURE 1:**
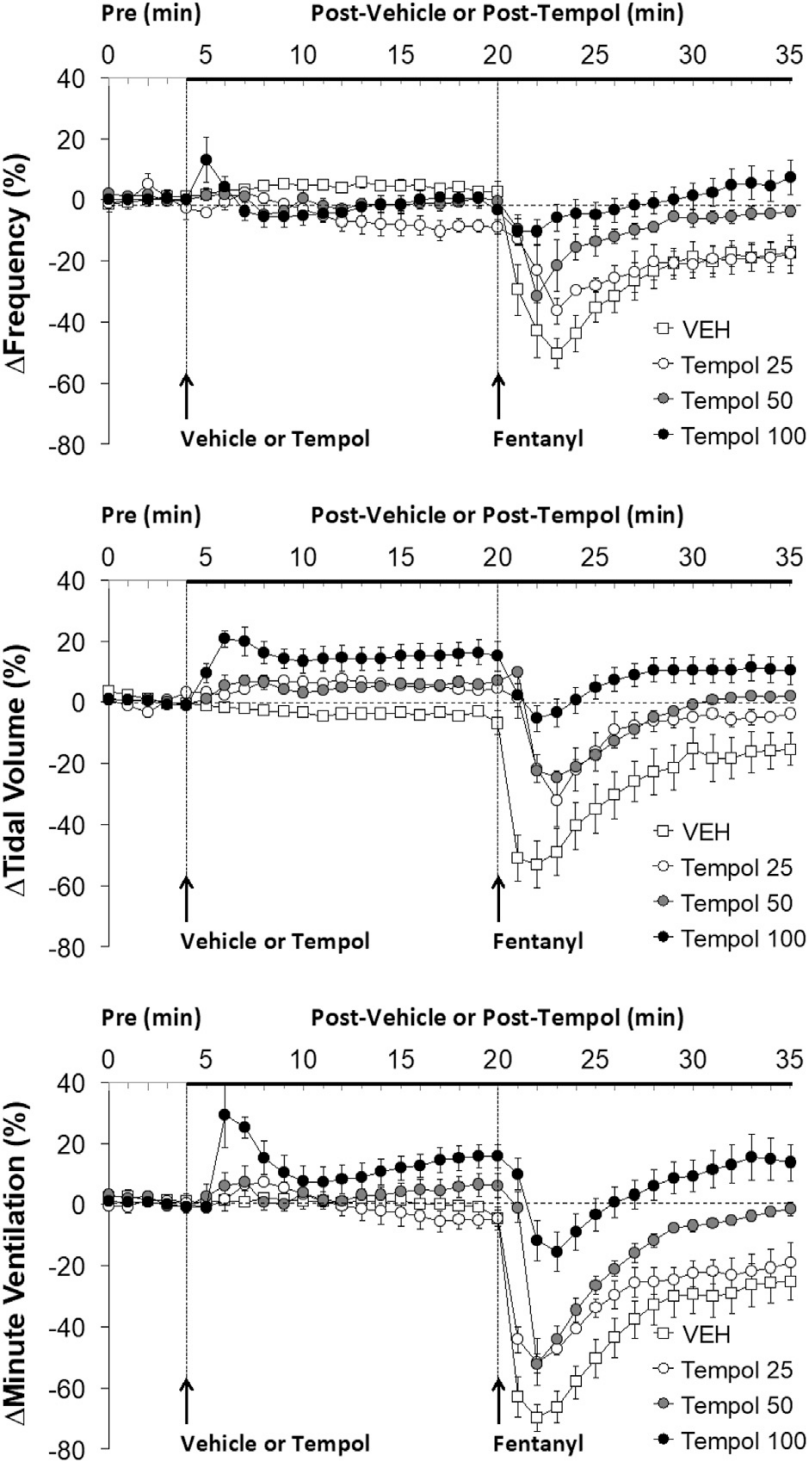
The changes in ventilatory parameters elicited by bolus injections of vehicle (VEH) or Tempol (25, 50 or 100 mg/kg, IV) and subsequent injections of fentanyl (5 μg/kg, IV) in isoflurane-anesthetized rats. There were 12 rats in the vehicle group, and 3, 5 and 12 rats in the 25, 50 and 100 mg/kg Tempol groups, respectively. The data presented are mean ± SEM.

**FIGURE 2:**
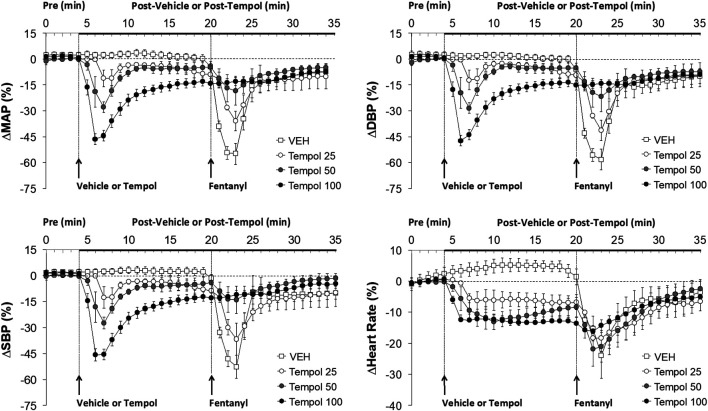
The changes in mean (MAP), diastolic (DBP) and systolic (SBP) arterial blood pressures and heart rate elicited by bolus injections of vehicle (VEH) or Tempol (25, 50 or 100 mg/kg, IV) and subsequent injections of fentanyl (5 μg/kg, IV) in isoflurane-anesthetized rats. There were 12 rats in the vehicle group, and 3, 5 and 12 rats in the 25, 50 and 100 mg/kg Tempol groups, respectively. The data presented are mean ± SEM.

**FIGURE 3:**
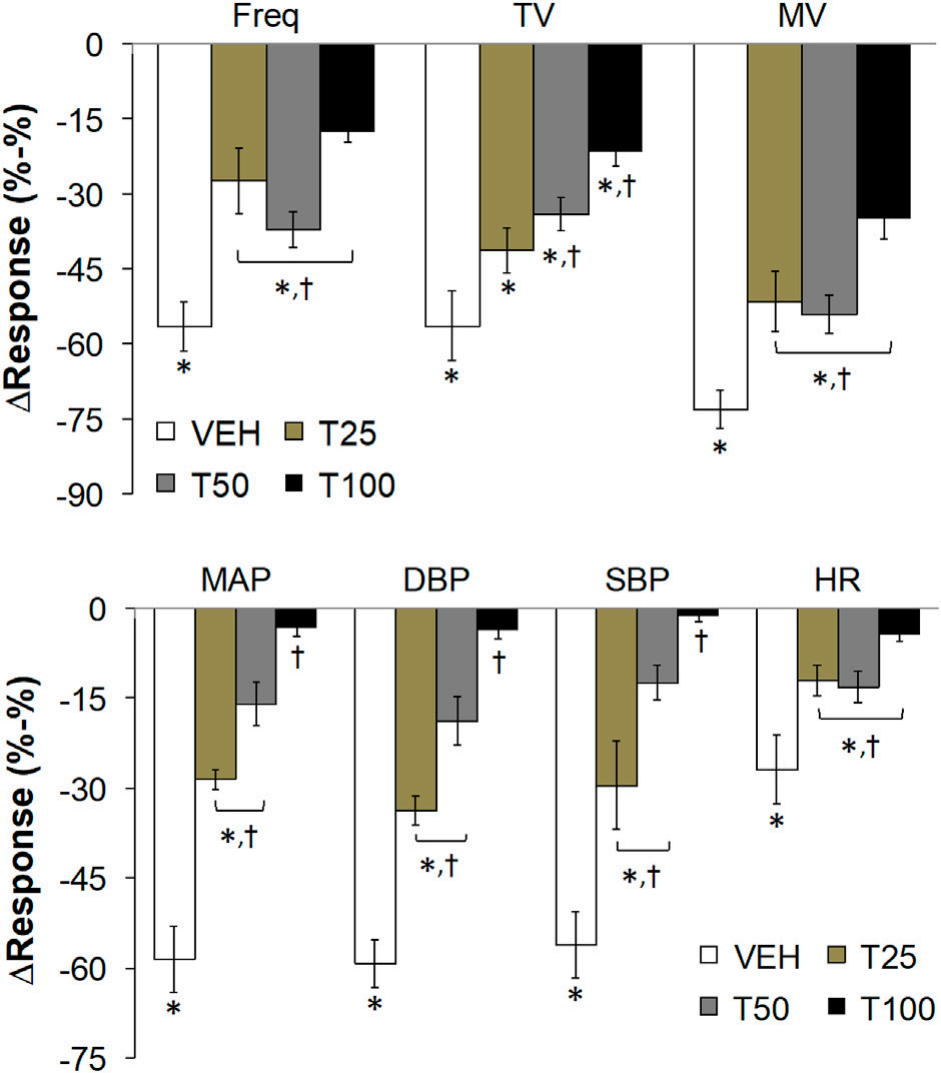
The peak changes in ventilatory parameters (top panel) and cardiovascular parameters (bottom panel) elicited by bolus injections of vehicle (VEH) or Tempol (25, 50 or 100 mg/kg, IV, designated T25, T50 and T100) and subsequent injections of fentanyl (5 μg/kg, IV) in isoflurane-anesthetized rats. There were 12 rats in the vehicle group, and 3, 5 and 12 rats in the 25, 50 and 100 mg/kg Tempol-treated groups, respectively. Data presented are mean ± SEM. *p < 0.05, significant change. ^†^P < T25, T50 and/or T100 vs. vehicle.

The baseline values recorded for the four groups of rats that eventually received vehicle or Tempol (25, 50 or 100 mg/kg, IV) are summarized in Supplementary Table S1. These data are presented as the mean ± SEM of the individual values recorded over a 5 min period. As can be seen, there were no between group differences for any parameter (*p* > 0.05 for all comparisons). All values in Figures 1–3 beginning at time zero (0) are expressed as the %change from the average pre-value for each parameter shown in Supplementary Table S1. All values in Supplementary Figures S4, S5 represent the sum of the individual responses (expressed as %change from pre-values shown in Supplementary Table S1) recorded at all time-points during each phase.

### Effects of Tempol Pretreatment on Fentanyl-Induced Decreases in Frequency of Breathing, Tidal Volume and Minute Ventilation

Data showing that bolus injections of Tempol (25, 50 or 100 mg/kg, IV) elicited minimal changes in the frequency of breathing (Freq) and somewhat dose-dependent increases in tidal volume (TV) and minute ventilation (MV) are summarized in Figure 1. The key finding was that a subsequent injection of fentanyl (5 μg/kg, IV) in these rats elicited profound decreases in Freq, TV and MV in vehicle-treated rats but progressively and substantially smaller responses as the dose of Tempol was increased. Indeed, the fentanyl-induced change in MV in the rats treated with the 100 mg/kg dose of Tempol returned to the elevated levels seen prior to injection of fentanyl. An injection of fentanyl (5 μg/kg, IV) markedly depressed the ventilatory waveform in a saline-treated rat but elicited progressively smaller responses in rats pre-injected with 25, 50 or 100 mg/kg of Tempol (each trace from separate rats) (Supplementary Figure S2).

### Effects of Tempol Pretreatment on Fentanyl-Induced Decreases in Arterial Blood Pressures

Data showing that injections of Tempol (25, 50 or 100 mg/kg, IV) elicited pronounced dose-dependent decreases in mean (MAP), diastolic (DBP) and systolic (SBP) arterial blood pressures that were still sustained for the 100 mg/kg dose at the time fentanyl was injected (16 min post-Tempol) are summarized in Figure 2. Tempol elicited minor but sustained decreases in heart rate (HR). As can be seen, the decreases in MAP, DBP and SBP were markedly diminished by Tempol, with the responses particularly reduced in size after administration of the 50 and 100 mg/kg doses of Tempol. The injection of fentanyl (5 μg/kg, IV) markedly depressed arterial blood pressure (SBP and DBP) in a saline-treated rat but elicited progressively smaller responses in rats pre-injected with 25, 50 or 100 mg/kg of Tempol (each trace from separate rats) (Supplementary Figure S3).

### Effects of Tempol Pretreatment on the Peak Changes in Cardiorespiratory Parameters Elicited by the Injection of Fentanyl

The peak changes in cardiorespiratory parameters elicited by fentanyl (5 μg/kg, IV) in vehicle or Tempol-treated rats are summarized in Figure 3. Tempol markedly diminished the peak decreases in all of these parameters. A summary of the total ventilatory responses (all time points summed together) elicited by Vehicle or Tempol and the subsequent injections of fentanyl are summarized in Supplementary Figure S4. Pre-values (5 min) did not change compared to the prior baseline recordings (top panel). Tempol (25, 50 or 100 mg/kg, IV; designated T25, T50, T100) elicited significant increases in TV and MV but not Freq (middle panel). The fentanyl-induced decreases in Freq, TV and MV were substantially blunted by Tempol (bottom panel). A summary of the total cardiovascular responses (all time points summed together) elicited by Vehicle or Tempol and the subsequent injections of fentanyl are summarized in Supplementary Figure S5. The pre-values (5 min) did not change compared to the prior baseline recordings (top panel). Tempol (25, 50 or 100 mg/kg, IV; designated T25, T50, T100) elicited significant decreases in MAP, DBP, SBP and HR (middle panel). The fentanyl-induced decreases in MAP, DBP and SBP (but not HR) were substantially blunted by Tempol (bottom panel).

### Effects of Tempol Pretreatment on Fentanyl-Induced Analgesia

**FIGURE 4:**
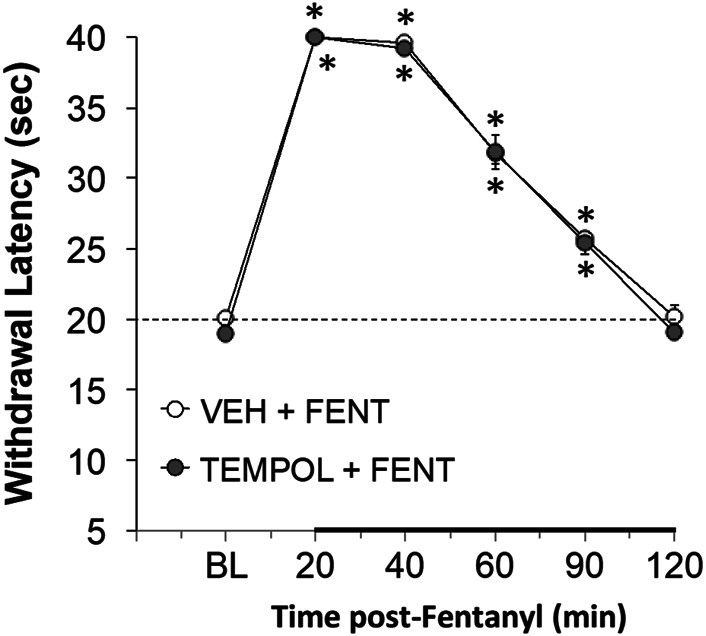
The changes in paw-withdrawal latency from baseline (BL) elicited by a bolus injection of fentanyl (25 μg/kg, IV) in conscious rats that had received an injection of vehicle (VEH) or Tempol (100 mg/kg, IV) 20 min previously. There were six rats in each group. The data are presented as mean ± SEM. *p < 0.05, significant from baseline. There were no differences between Vehicle- or Tempol-treated rats at any time point (*p* > 0.05, for all comparisons).

With respect to analgesia, we found that Tempol at various doses (1 mg/kg, *n* = 4; 10 mg/kg, *n* = 4, or 100 mg/kg, *n* = 6) had no effects on paw-withdrawal latencies of conscious rats for up to 2 h post-administration (data not shown). The analgesic effects (increases in paw-withdrawal latency) in conscious rats elicited by a bolus injection of fentanyl (25 μg/kg, IV) were not affected by pretreatment with Tempol (100 mg/kg, IV) at a study commenced at 10 AM (see Figure 4). Fentanyl given to the same rats at 4 PM (no drug pretreatments at this time) elicited significant analgesia of much reduced duration (Supplementary Figure S6 **)**. The injection of Tempol in the 10 AM study did not affect this loss of analgesic effect of fentanyl at 4 PM.

### Effects of L-NACme Pretreatment on Fentanyl-Induced Analgesia

Pretreatment with a large IV dose of L-NACme (500 μmol/kg, 88.61 mg/kg) had minimal effects on the ventilatory depressant effects elicited by a subsequent injection of fentanyl (25 μg/kg, IV) (Supplementary Figure S7). The 10 AM injection of L-NACme substantially blunted the development of tolerance to the analgesic responses elicited by the second injection of fentanyl given at 4 PM (Supplementary Figure S8).

## Discussion

Novel findings of this study in isoflurane-anesthetized rats were that 1) the systemic injection of Tempol elicited positive effects on MV in naïve rats, 2) Tempol pretreatment dose-dependently and markedly attenuated the cardiorespiratory depressant effects elicited by injection of fentanyl, and 3) injection of Tempol immediately reversed the pronounced cardiorespiratory depression elicited by an infusion of fentanyl. Other key findings were that in conscious rats, 1) Tempol did not affect fentanyl-induced analgesia or 2) modify the decline in analgesia elicited by a second dose of fentanyl in the same rats. The ability of Tempol to profoundly affect the cardiorespiratory depressant effects of fentanyl without modulating fentanyl-induced analgesia would therefore seem to be by mechanisms that are independent of direct effects on ORs.

The obvious question pertains to the mechanism(s) by which Tempol exerts its positive affect against fentanyl. It is tempting to assume that Tempol attenuates the respiratory depressant effects of fentanyl by scavenging free radicals such as superoxide anion (Wilcox and Pearlman, 2008; Wilcox, 2010; Kim et al., 2016; Bernardy et al., 2017; Wang et al., 2018; Chiarotto et al., 2019; Afjal et al., 2019) although available data showed that fentanyl and analogues reduce (Kim et a., 2017) or have no effect (Krumholz et al., 1993; Jaeger et al., 1998) on oxidative stress although high-dose remifentanil increases myocardial oxidative stress (Mei et al., 2013). However, this may be an important feature of Tempol since a search of the literature found that microinjection of Tempol into the nucleus accumbens blocks expression of morphine conditioned place preference in rats (Qi et al., 2015). Our own data question this possibility with respect to fentanyl-induced cardiorespiratory depression, since 1) Tempol did not modify development of tolerance to the analgesic actions of fentanyl in conscious rats (present study), 2) scavenging free radicals with D-penicillamine markedly attenuated development of tolerance to the respiratory depressant effects of morphine in freely moving rats (Young et al., 2013), and 3) that L-NACme, a powerful reducing agent (Michaelsen et al., 2009; Giustarini et al., 2012; Poopari et al., 2015; Uemura et al., 2018; Giustarini et al., 2018), diminished development of tolerance to the analgesic actions of fentanyl in conscious rats (present study). Taken together, it would seem possible that Tempol directly interferes (i.e., independently of superoxide/free radical scavenging) with intracellular signaling processes by which fentanyl elicits its cardiorespiratory depressant effects.

Despite compelling evidence that Tempol acts via scavenging superoxide anion and free radicals, Xu et al. (2004) reported that Tempol lowers mean arterial blood pressure (MAP) and sympathetic nerve activity in rats by mechanisms other than scavenging these radicals. As key background, NAD(P)H oxidase is the major generator of O^2−^ in tissues (Rajagopalan et al., 1996; Griendling et al., 2000; Beswick et al., 2001; Lassègue and Clempus, 2003; Ulker et al., 2003) and apocynin, is an NAD(P)H oxidase inhibitor that reduces O^2−^ production *in vitro* and *in vivo* (Hamilton et al., 2001; Hamilton et al., 2002; Ulker et al., 2003). In addition, polyethylene glycol-superoxide dismutase (PEG-SOD) is a potent membrane-permeable SOD analog that protects against myocardial ischemia-reperfusion injury by reducing O^2−^ levels (Schleien et al., 1994; Nguyen et al., 1999). In their study, Xu et al. (2004) tested the hypothesis that the depressor responses caused by Tempol are not due to reductions in vascular O_2_^−^ levels in urethane-anesthetized deoxy-corticosterone acetate (DOCA)-salt hypertensive rats. They compared the effects of intravenous (IV) Tempol, apocynin, PEG-SOD, and SOD on MAP, heart rate, and renal sympathetic nerve activity (RSNA). In DOCA-salt rats, Tempol (30–300 μmol/kg, 5–516 mg/kg) dose-dependently decreased RSNA, MAP, and heart rate. However, Tempol did not reduce O^2−^ levels (dihydroethidium-induced fluorescent signals) in the aorta and vena cava of these rats. In addition, Apocynin (200 μmol/kg) did not lower MAP or heart rate and apocynin did not potentiate depressor responses caused by Tempol. Moreover, PEG-SOD (10,000 U/kg, bolus or 5000 U/kg bolus followed by a 30 min infusion of 500 U/kg/min) or SOD (25,000 U/kg, bolus or 10,000 U/kg bolus followed by a 30 min infusion of 1000 U/kg/min) did not alter MAP or heart rate. Xu et al. (2004) concluded that the depressor responses and decreases in heart rate and RSNA caused by acute Tempol treatment are caused by molecular mechanisms that are independent of any potential SOD-mimetic action.

Several pharmacological agents have been developed to target both central and peripheral chemoreceptors to mitigate opioid-induced respiratory depression (OIRD) via activation of non-opioidergic pathways without comprising opioid-induced analgesia (Dahan et al., 2010). We have identified a peripheral chemoreceptor stimulant, GAL-021, that in preclinical (Dallas et al., 2015; Golder et al., 2015) and experimental human studies (McLeod et al., 2014; Roozekrans et al., 2014) augmented ventilation and reversed OIRD without compromising opioid-induced analgesia. The data strongly suggested that these effects of GAL-021 were due to blocking Ca^2+^-activated potassium channels (BKCa) in peripheral carotid body chemoreceptor (glomus) cells (McLeod et al., 2014; Roozekrans et al., 2014; Dallas et al., 2015; Golder et al., 2015). As such, it would seem feasible that the ability of Tempol to blunt the ventilatory effects of fentanyl may involve the *blockade* of BKCa channels. However, it has been established that Tempol elicits profound vasodilation by the direct activation BKCa channels in vascular smooth muscle (Xu et al., 2005, Xu et al., 2006). As such, the ability of Tempol to block BKCa channels may contribute to the hypotensive effects of this drug but it therefore seems unlikely that this is the mechanism that blocks the hypotensive effects or by analogy, the respiratory depressant effects of fentanyl.

## Study Limitations

This study provides compelling evidence that pretreatment of isoflurane-anesthetized rats with Tempol markedly blunts the cardiorespiratory depressant effects elicited by subsequent injection of fentanyl. Pretreatment strategies in anesthetized subjects are vital in clinical scenarios such as in the operating room when a drug with pharmacological profile designed to prevent the negative cardiorespiratory actions of opioids without compromising analgesia is administered before or in combination with the opioid. However, the present study is limited in several ways and these imitations will be addressed in future studies in which we will 1) inject Tempol prior to fentanyl in freely moving rats to eliminate the potential confounding influence of anesthesia and to gain information as to whether Tempol modifies the behavioral (e.g., sedative) effects of fentanyl, 2) inject selected doses of Tempol after the injection of high doses of fentanyl in anesthetized and freely moving rats to determine the efficacy of Tempol as a reversal agent that would be impactful in clinical (e.g., post-surgical) scenarios and in emergency situations to overcome opioid-induced overdose in the setting of the general population.

At present, we do not know the principal mechanisms by which Tempol blunts fentanyl-induced cardiorespiratory depression other than suspecting it is unlikely that they involve direct interaction with opioid receptors (since Tempol spares analgesia) or BK_Ca_ channels (see discussion above). With respect to evaluating the protein targets and mechanisms of action of Tempol, we working with Dr. Christopher Ellis (US Army, DEVCOM Chemical Biological Center) who will use Public Health Assessment via Structural Evaluation (PHASE) (Ellis et al., 2019) and molecular docking methods (Ellis et al., 2018) to identify protein sites (e.g., ion-channels, Gprotein-coupled receptors, membrane-bound and intracellular enzymes) to which Tempol, morphine and fentanyl bind with specific focus on signaling pathways relevant to opioid-induced respiratory depression (Ellis et al., 2018). The first step will develop the binding profiles for each drug of interest, and identify the potential biological targets and mode(s) of action by using PHASE. These computational studies will develop a ranked list of potential mechanisms of action for each compound of interest along with structurally similar analogs. The second step will use molecular docking models to predict the binding affinity of the drugs at the primary and secondary targets identified in the first step.

## Conclusion

In summary, we found that Tempol efficiently prevents fentanyl-induced depression of both breathing and arterial blood pressure in male rats. Although we did not establish that the effects of Tempol were due to its known ability to scavenge superoxide anion, our data do suggest that Tempol, which is a clinically approved drug for the treatment of alopecia (Wilcox and Pearlman, 2008; Wilcox, 2010) and related Tempol structures (see Supplementary Figure S9) may be repurposed as an intravenous agent to protect/reverse the negative cardiorespiratory effects of fentanyl while preserving analgesia in male and female children and adults. Future work on elucidating Tempol’s effects on fentanyl signaling should involve elucidation of Tempol’s known superoxide anion scavenging ability and investigation into Tempol interacts with proteins in the fentanyl cell-signaling pathway. We are continuing our studies by examining superoxide anion scavenger molecules with different chemical structures from Tempol, as well as performing high throughput screening methods such as surface plasmon resonance, hydrogen deuterium exchange mass spectrometry, and Isothermal titration calorimetry on proteins in the fentanyl signaling pathway (Marcsisin and Engen, 2010; Nguyen et al., 2015; Baranauskiene et al., 2019). This future work will characterize the molecular mechanisms by which Tempol is able to prevent fentanyl-induced depression of breathing and blood pressure without affecting analgesia.

## Data Availability

The raw data supporting the conclusions of this article will be made available by the authors, without undue reservation.

## Abbreviations

OR, opioid receptor; OIRD, opioid-induced respiratory depression; Tempol, 4-hydroxy-2,2,6,6-tetramethylpiperidine-N-oxyl; L-NACme, N-acetyl-L-cysteine methyl ester; BKCa, Ca2+-activated K+- channels; SOD, superoxide dismutase; Freq, frequency of breathing; TV, tidal volume; MV, minute ventilation; A-a gradient, Alveolar-arterial gradient; HR, heart rate; MAP, mean arterial blood pressure; SBP, systolic arterial blood pressure; DBP, diastolic arterial blood pressure; RSNA, renal sympathetic nerve activity; PEG-SOD, polyethylene glycol-superoxide dismutase; DOCA, deoxy-corticosterone acetate

## References

[B1] Afjal M. Abdi S. H. Sharma S. Ahmad S. Fatima M. Dabeer S. (2019). Anti-inflammatory Role of Tempol (4-Hydroxy-2,2,6,6-Tetramethylpiperidin-1-Oxyl) in Nephroprotection. Hum. Exp. Toxicol. 38, 713–723. 10.1177/0960327119836203 30924375

[B2] Almeida M. B. Costa-Malaquias A. Nascimento J. L. M. Oliveira K. R. Herculano A. M. Crespo-López M. E. (2014). Therapeutic Concentration of Morphine Reduces Oxidative Stress in Glioma Cell Line. Braz. J. Med. Biol. Res. 47, 398–402. 10.1590/1414-431x20143697 24728211 PMC4075308

[B3] Armenian P. Vo K. T. Barr-Walker J. Lynch K. L. (2018). Fentanyl, Fentanyl Analogs and Novel Synthetic Opioids: A Comprehensive Review. Neuropharmacology 134, 121–132. 10.1016/j.neuropharm.2017.10.016 29042317

[B4] Baby S. M. Gruber R. B. Young A. P. MacFarlane P. M. Teppema L. J. Lewis S. J. (2018). Bilateral Carotid Sinus Nerve Transection Exacerbates Morphine-Induced Respiratory Depression. Eur. J. Pharmacol. 834, 17–29. 10.1016/j.ejphar.2018.07.018 30012498 PMC6091892

[B5] Baranauskiene L. Kuo T.-C. Chen W.-Y. Matulis D. (2019). Isothermal Titration Calorimetry for Characterization of Recombinant Proteins. Curr. Opin. Biotechnol. 55, 9–15. 10.1016/j.copbio.2018.06.003 30031160

[B6] Bernardy C. C. F. Zarpelon A. C. Pinho-Ribeiro F. A. Calixto-Campos C. Carvalho T. T. Fattori V. (2017). Tempol, a Superoxide Dismutase Mimetic Agent, Inhibits Superoxide Anion-Induced Inflammatory Pain in Mice. Biomed. Res. Int. 2017, 1–15. 10.1155/2017/9584819 PMC5446866 28589150

[B7] Beswick R. A. Dorrance A. M. Leite R. Webb R. C. (2001). NADH/NADPH Oxidase and Enhanced Superoxide Production in the Mineralocorticoid Hypertensive Rat. Hypertension 38, 1107–1111. 10.1161/hy1101.093423 11711506

[B9] Chiarotto G. B. Cartarozzi L. P. Perez M. Biscola N. P. Spejo A. B. Gubert F. (2019). Tempol Improves Neuroinflammation and Delays Motor Dysfunction in a Mouse Model (SOD1G93A) of ALS. J. Neuroinflammation 16, 218. 10.1186/s12974-019-1598-x 31727149 PMC6857328

[B11] Dahan A. Aarts L. Smith T. W. (2010). Incidence, Reversal, and Prevention of Opioid-Induced Respiratory Depression. Anesthesiology 112, 226–238. 10.1097/ALN.0b013e3181c38c25 20010421

[B12] Dahan A. van der Schrier R. Smith T. Aarts L. van Velzen M. Niesters M. (2018). Averting Opioid-Induced Respiratory Depression without Affecting Analgesia. Anesthesiology 128, 1027–1037. 10.1097/ALN.0000000000002184 29553984

[B13] Dallas M. L. Peers C. Golder F. J. Baby S. Gruber R. MacIntyre D. E. (2015). GAL-021 and GAL-160 Are Efficacious in Rat Models of Obstructive and Central Sleep Apnea and Inhibit BKCa in Isolated Rat Carotid Body Glomus Cells. Adv. Exp. Med. Biol. 860, 361–370. 10.1007/978-3-319-18440-1_41 26303501

[B14] Ellis C. R. Kruhlak N. L. Kim M. T. Hawkins E. G. Stavitskaya L. (2018). Predicting Opioid Receptor Binding Affinity of Pharmacologically Unclassified Designer Substances Using Molecular Docking. PLoS One 13, e0197734. 10.1371/journal.pone.0197734 29795628 PMC5967713

[B15] Ellis C. R. Racz R. Kruhlak N. L. Kim M. T. Hawkins E. G. Strauss D. G. (2019). Assessing the Structural and Pharmacological Similarity of Newly Identified Drugs of Abuse to Controlled Substances Using Public Health Assessment via Structural Evaluation. Clin. Pharmacol. Ther. 106, 116–122. 10.1002/cpt.1418 30957872 PMC6617983

[B16] Gharagozlou P. Hashemi E. DeLorey T. Clark J. D. Lameh J. (2006). Pharmacological Profiles of Opioid Ligands at Kappa Opioid Receptors. BMC Pharmacol. 6, 3. 10.1186/1471-2210-6-3 16433932 PMC1403760

[B17] Giustarini D. Galvagni F. Dalle Donne I. Milzani A. Severi F. M. Santucci A. (2018). N-acetylcysteine Ethyl Ester as GSH Enhancer in Human Primary Endothelial Cells: A Comparative Study with Other Drugs. Free Radic. Biol. Med. 126, 202–209. 10.1016/j.freeradbiomed.2018.08.013 30114478

[B18] Giustarini D. Milzani A. Dalle-Donne I. Tsikas D. Rossi R. (2012). N-Acetylcysteine Ethyl Ester (NACET): a Novel Lipophilic Cell-Permeable Cysteine Derivative with an Unusual Pharmacokinetic Feature and Remarkable Antioxidant Potential. Biochem. Pharmacol. 84, 1522–1533. 10.1016/j.bcp.2012.09.010 23000913

[B19] Golder F. J. Dax S. Baby S. M. Gruber R. Hoshi T. Ideo C. (2015). Identification and Characterization of GAL-021 as a Novel Breathing Control Modulator. Anesthesiology 123, 1093–1104. 10.1097/ALN.0000000000000844 26352381

[B20] Griendling K. K. Sorescu D. Ushio-Fukai M. (2000). NAD(P)H Oxidase. Circ. Res. 86, 494–501. 10.1161/01.res.86.5.494 10720409

[B21] Hamilton C. A. Brosnan M. J. Al-Benna S. Berg G. Dominiczak A. F. (2002). NAD(P)H Oxidase Inhibition Improves Endothelial Function in Rat and Human Blood Vessels. Hypertension 40, 755–762. 10.1161/01.hyp.0000037063.90643.0b 12411473

[B22] Hamilton C. A. Brosnan M. J. McIntyre M. Graham D. Dominiczak A. F. (2001). Superoxide Excess in Hypertension and Aging. Hypertension 37, 529–534. 10.1161/01.hyp.37.2.529 11230330

[B23] Hargreaves K. Dubner R. Brown F. Flores C. Joris J. (1988). A New and Sensitive Method for Measuring thermal Nociception in Cutaneous Hyperalgesia. Pain 32, 77–88. 10.1016/0304-3959(88)90026-7 3340425

[B24] Henderson F. May W. J. Gruber R. B. Young A. P. Palmer L. A. Gaston B. (2013). Low-dose Morphine Elicits Ventilatory Excitant and Depressant Responses in Conscious Rats: Role of Peripheral μ-opioid Receptors. Open J. Mol. Integr. Physiol. 3, 111–124. 10.4236/ojmip.2013.33017 24900948 PMC4041292

[B25] Henderson F. May W. J. Gruber R. B. Discala J. F. Puskovic V. Young A. P. (2014). Role of central and Peripheral Opiate Receptors in the Effects of Fentanyl on Analgesia, Ventilation and Arterial Blood-Gas Chemistry in Conscious Rats. Respir. Physiol. Neurobiol. 191, 95–105. 10.1016/j.resp.2013.11.005 24284037 PMC4391496

[B26] Jaeger K. Scheinichen D. Heine J. André M. Bund M. Piepenbrock S. (1998). Remifentanil, Fentanyl, and Alfentanil Have No Influence on the Respiratory Burst of Human Neutrophilsin Vitro. Acta Anaesthesiol. Scand. 42, 1110–1113. 10.1111/j.1399-6576.1998.tb05386.x 9809098

[B27] Kim C. H. Jeong S. S. Yoon J. Y. Yoon J. U. Yu S. B. Kim E. J. (2017). Remifentanil Reduced the Effects of Hydrogen Peroxide-Induced Oxidative Stress in Human Keratinocytes via Autophagy. Connect. Tissue Res. 58, 597–605. 10.1080/03008207.2017.1285915 28165802

[B28] Kim H. K. Hwang S.-H. Abdi S. (2016). Tempol Ameliorates and Prevents Mechanical Hyperalgesia in a Rat Model of Chemotherapy-Induced Neuropathic Pain. Front. Pharmacol. 07, 532. 10.3389/fphar.2016.00532 PMC5237846 28138318

[B29] Krumholz W. Demel C. Jung S. Meuthen G. Hempelmann G. (1993). The Influence of Fentanyl and Alfentanil on Functions of Human Polymorphonuclear Leukocytes *In Vitro* . Acta Anaesthesiol. Scand. 37, 386–389. 10.1111/j.1399-6576.1993.tb03734.x 8391745

[B30] Lassègue B. Clempus R. E. (2003). Vascular NAD(P)H Oxidases: Specific Features, Expression, and Regulation. Am. J. Physiology-Regulatory, Integr. Comp. Physiol. 285, R277–R297. 10.1152/ajpregu.00758.2002 12855411

[B31] Lee J. Kim M. S. Park C. Jung E. B. Choi D. H. Kim T. Y. (2004). Morphine Prevents Glutamate‐Induced Death of Primary Rat Neonatal Astrocytes Through Modulation of Intracellular Redox. Immunopharmacol. Immunotoxicol. 26, 17–28. 10.1081/iph-120029941 15106729

[B32] Leventelis C. Goutzourelas N. Kortsinidou A. Spanidis Y. Toulia G. Kampitsi A. (2019). Buprenorphine and Methadone as Opioid Maintenance Treatments for Heroin-Addicted Patients Induce Oxidative Stress in Blood. Oxidative Med. Cell Longevity 2019, 1–9. 10.1155/2019/9417048 PMC6481042 31093318

[B33] Lipiński P. F. J. Kosson P. Matalińska J. Roszkowski P. Czarnocki Z. Jarończyk M. (2019). Fentanyl Family at the Mu-Opioid Receptor: Uniform Assessment of Binding and Computational Analysis. Molecules 24, E740. 10.3390/molecules24040740 30791394 PMC6412969

[B34] Marcsisin S. R. Engen J. R. (2010). Hydrogen Exchange Mass Spectrometry: what Is it and what Can it Tell Us?. Anal. Bioanal. Chem. 397, 967–972. 10.1007/s00216-010-3556-4 20195578 PMC2868954

[B35] May W. J. Gruber R. B. Discala J. F. Puskovic V. Henderson F. Palmer L. A. (2013b). Morphine Has Latent Deleterious Effects on the Ventilatory Responses to a Hypoxic challenge. Open J. Mol. Integr. Physiol. 3, 166–180. 10.4236/ojmip.2013.34022 25045593 PMC4103751

[B36] May W. J. Henderson Jr. F. Gruber R. B. Discala J. F. Young A. P. Bates J. N. (2013a). Morphine Has Latent Deleterious Effects on the Ventilatory Responses to a Hypoxic-Hypercapnic challenge. Ojmip 03, 134–145. 10.4236/ojmip.2013.33019 PMC4103749 25045592

[B37] Mayer N. Zimpfer M. Raberger G. Beck A. (1989). Fentanyl Inhibits the Canine Carotid Chemoreceptor Reflex. Anesth. Analgesia 69, 756–762. 10.1213/00000539-198912000-00012 2589656

[B38] McLeod J. F. Leempoels J. M. Peng S. X. Dax S. L. Myers L. J. Golder F. J. (2014). GAL-021, a New Intravenous BK Ca -channel Blocker, Is Well Tolerated and Stimulates Ventilation in Healthy Volunteers. Br. J. Anaesth. 113, 875–883. 10.1093/bja/aeu182 24989775

[B39] Mei B. Wang T. Wang Y. Xia Z. Irwin M. G. Wong G. T. C. (2013). High Dose Remifentanil Increases Myocardial Oxidative Stress and Compromises Remifentanil Infarct-Sparing Effects in Rats. Eur. J. Pharmacol. 718, 484–492. 10.1016/j.ejphar.2013.07.030 23954793

[B40] Michaelsen J. T. Dehnert S. Giustarini D. Beckmann B. Tsikas D. (2009). HPLC Analysis of Human Erythrocytic Glutathione Forms Using OPA and N-Acetyl-Cysteine Ethyl Ester: Evidence for Nitrite-Induced GSH Oxidation to GSSG. J. Chromatogr. B 877, 3405–3417. 10.1016/j.jchromb.2009.06.043 19665947

[B41] Motaghinejad M. Karimian M. Motaghinejad O. Shabab B. Yazdani I. Fatima S. (2015). Protective Effects of Various Dosage of Curcumin against Morphine Induced Apoptosis and Oxidative Stress in Rat Isolated hippocampus. Pharmacol. Rep. 67, 230–235. 10.1016/j.pharep.2014.09.006 25712644

[B42] Nguyen H. Park J. Kang S. Kim M. (2015). Surface Plasmon Resonance: a Versatile Technique for Biosensor Applications. Sensors 15, 10481–10510. 10.3390/s150510481 25951336 PMC4481982

[B43] Nguyen W. D. Kim D. H. Alam H. B. Provido H. S. Kirkpatrick J. R. (1999). Polyethylene Glycol- Superoxide Dismutase Inhibits Lipid Peroxidation in Hepatic Ischemia/reperfusion Injury. Crit. Care 3, 127–130. 10.1186/cc358 11056736 PMC29026

[B44] Poopari M. R. Dezhahang Z. Xu Y. (2015). Identifying Dominant Conformations of N-Acetyl-L-Cysteine Methyl Ester and N-Acetyl-L-Cysteine in Water: VCD Signatures of the Amide I and the CO Stretching Bands. Spectrochimica Acta A: Mol. Biomol. Spectrosc. 136, 131–140. 10.1016/j.saa.2013.08.118 24076069

[B45] Qi C. Wang X. Ge F. Li Y. Shen F. Wang J. (2015). mGluR5 in the Nucleus Accumbens Shell Regulates Morphine-Associated Contextual Memory through Reactive Oxygen Species Signaling. Addict. Biol. 20, 927–940. 10.1111/adb.12222 25736529

[B46] Rajagopalan S. Kurz S. Münzel T. Tarpey M. Freeman B. A. Griendling K. K. (1996). Angiotensin II-Mediated Hypertension in the Rat Increases Vascular Superoxide Production via Membrane NADH/NADPH Oxidase Activation. Contribution to Alterations of Vasomotor Tone. J. Clin. Invest. 97, 1916–1923. 10.1172/JCI118623 8621776 PMC507261

[B47] Raynor K. Kong H. Chen Y. Yasuda K. Yu L. Bell G. I. (1994). Pharmacological Characterization of the Cloned Kappa-, delta-, and Mu-Opioid Receptors. Mol. Pharmacol. 45, 330–334. 8114680

[B48] Roozekrans M. van der Schrier R. Okkerse P. Hay J. McLeod J. F. Dahan A. (2014). Two Studies on Reversal of Opioid-Induced Respiratory Depression by BK-Channel Blocker GAL021 in Human Volunteers. Anesthesiology 121, 459–468. 10.1097/ALN.0000000000000367 25222672

[B49] Schleien C. L. Eberle B. Shaffner D. H. Koehler R. C. Traystman R. J. (1994). Reduced Blood-Brain Barrier Permeability after Cardiac Arrest by Conjugated Superoxide Dismutase and Catalase in Piglets. Stroke 25, 1830–1834. 10.1161/01.str.25.9.1830 8073465

[B50] Skrabalova J. Karlovska I. Hejnova L. Novotny J. (2018). Protective Effect of Morphine Against the Oxidant-Induced Injury in H9c2 Cells. Cardiovasc. Toxicol. 18, 374–385. 10.1007/s12012-018-9448-0 29380194

[B51] Suzuki J. El-Haddad S. (2017). A Review: Fentanyl and Non-pharmaceutical Fentanyls. Drug and Alcohol Dependence 171, 107–116. 10.1016/j.drugalcdep.2016.11.033 28068563

[B52] Uemura T. Watanabe K. Ko K. Higashi K. Kogure N. Kitajima M. (2018). Protective Effects of Brain Infarction by N -Acetylcysteine Derivatives. Stroke 49, 1727–1733. 10.1161/STROKEAHA.118.021755 29866754

[B53] Ulker S. Mckeown P. P. Bayraktutan U. (2003). Vitamins Reverse Endothelial Dysfunction through Regulation of eNOS and NAD(P)H Oxidase Activities. Hypertension 41, 534–539. 10.1161/01.HYP.0000057421.28533.37 12623955

[B54] Wallenstein S. Zucker C. L. Fleiss J. L. (1980). Some Statistical Methods Useful in Circulation Research. Circ. Res. 47, 1–9. 10.1161/01.res.47.1.1 7379260

[B55] Wang Y. Hai B. Ai L. Cao Y. Li R. Li H. (2018). Tempol Relieves Lung Injury in a Rat Model of Chronic Intermittent Hypoxia via Suppression of Inflammation and Oxidative Stress. Iran J. Basic Med. Sci. 21, 1238–1244. 10.22038/ijbms.2018.31716.7714 30627367 PMC6312670

[B56] Wilcox C. S. (2010). Effects of Tempol and Redox-Cycling Nitroxides in Models of Oxidative Stress. Pharmacol. Ther. 126, 119–145. 10.1016/j.pharmthera.2010.01.003 20153367 PMC2854323

[B57] Wilcox C. S. Pearlman A. (2008). Chemistry and Antihypertensive Effects of Tempol and Other Nitroxides. Pharmacol. Rev. 60, 418–469. 10.1124/pr.108.000240 19112152 PMC2739999

[B58] Xu H. Bian X. Watts S. W. Hlavacova A. (2005). Activation of Vascular BK Channel by Tempol in DOCA-Salt Hypertensive Rats. Hypertension 46, 1154–1162. 10.1161/01.HYP.0000186278.5027510.1161/01.hyp.0000186278.50275.fa 16216988

[B59] Xu H. Fink G. D. Galligan J. J. (2004). Tempol Lowers Blood Pressure and Sympathetic Nerve Activity But Not Vascular O 2 − in DOCA-Salt Rats. Hypertension 43, 329–334. 10.1161/01.HYP.0000112304.26158.5c 14707156

[B60] Xu H. Jackson W. F. Fink G. D. Galligan J. J. (2006). Activation of Potassium Channels by Tempol in Arterial Smooth Muscle Cells from Normotensive and Deoxycorticosterone Acetate-Salt Hypertensive Rats. Hypertension 48, 1080–1087. 10.1161/01.HYP.0000249511.96555.57 17060504

[B61] Yeadon M. Kitchen I. (1990). Multiple Opioid Receptors Mediate the Respiratory Depressant Effects of Fentanyl-like Drugs in the Rat. Gen. Pharmacol. Vasc. Syst. 21, 655–664. 10.1016/0306-3623(90)91013-h 2177434

[B62] Young A. P. Gruber R. B. Discala J. F. May W. J. McLaughlin D. Palmer L. A. (2013). Co-activation of μ- and δ-opioid Receptors Elicits Tolerance to Morphine-Induced Ventilatory Depression via Generation of Peroxynitrite. Respir. Physiol. Neurobiol. 186, 255–264. 10.1016/j.resp.2013.02.028 23473921 PMC4451624

[B63] Zhu J. Xue J.-C. Law P.-Y. Claude P. A. Luo L.-Y. Yin J. (1996). The Region in the μ Opioid Receptor Conferring Selectivity for Sufentanil over the δ Receptor Is Different from that over the κ Receptor. FEBS Letts 384, 198–202. 10.1016/0014-5793(96)00312-2 8612823

