# Supplementary material for: Systemic Administration of Tempol Attenuates the Cardiorespiratory Depressant Effects of Fentanyl
Source: Front Pharmacol. 2021 Jun 23;12:690407. doi: 10.3389/fphar.2021.690407 (PMC8260831; doi:10.3389/fphar.2021.690407)
Supplement: Supplementary file 1 [file DataSheet1.PDF]

Supplement

Systemic administration of Tempol attenuates the cardiorespiratory depressant effects of fentanyl

Santhosh M. Baby,[1,†] Ryan B. Gruber,[1] Joseph F. Discala,[1] Veljko Puskovic,[1]

Nijo Jose,[2] Feixiong Cheng,[3] Michael W. Jenkins,[4,5] James M. Seckler,[4] Stephen J. Lewis[5,*]

[1]Galleon Pharmaceuticals, Inc., 213 Witmer Road, Horsham, PA, USA

[2]Department of Radiotherapy and Oncology, Kasturba Medical College, Manipal, Karnataka, India

[3]Cleveland Clinic Lerner College of Medicine, Case Western Reserve University, Cleveland, OH, USA

[4]Department of Biomedical Engineering, Case Western Reserve University, Cleveland, OH, USA

[5]Department of Pediatrics, Case Western Reserve University, Cleveland, OH, USA

[†]**Current address:** Santhosh M. Baby, Translational Sciences Treatment Discovery, Galvani Bioelectronics, Inc., 1250 S Collegeville Rd., Collegeville, Pennsylvania 1r9426. Email: santhosh.m.baby@galvani.bio

***Corresponding Author:**
Stephen J. Lewis, PhD
Department of Pediatrics, Division of Pulmonology, Allergy and Immunology
Department of Pharmacology School of Medicine
Biomedical Research Building, Room 831
Case Western Reserve University
10900 Euclid Avenue
Cleveland, OH 44106-4984
Phone: 216-368-3482
Email: sjl78@case.edu

Supplemental Table 1. Baseline parameters

	Vehicle	Tempol (mg/kg, IV)		
**Parameter**	**Saline (0.1 ml/kg)**	**25**	**50**	**100**
Number of rats	12	3	5	12
Frequency, breaths/min	53 ± 2	62 ± 5	60 ± 3	58 ± 4
Tidal Volume, ml/min	2.1 ± 0.1	1.9 ± 0.2	2.1 ± 0.1	2.3 ± 0.2
Minute Ventilation, ml/min	108 ± 4	120 ± 14	125 ± 3	128 ± 9
MAP, mmHg	112 ± 3	118 ± 6	110 ± 11	117 ± 6
SBP, mmHg	152 ± 3	161 ± 10	142 ± 13	162 ± 7
DBP, mmHg	94 ± 2	97 ± 5	109 ± 13	99 ± 4
Heart rate, beats/min	340 ± 6	362 ± 15	354 ± 18	348 ± 14

MAP, mean arterial blood pressure; SBP, systolic arterial blood pressure; DBP, diastolic arterial blood pressure. The data are presented as mean ± SEM. There were no between group differences for any parameter ($P > 0.05$ for all comparisons).

Supplemental Figure 1

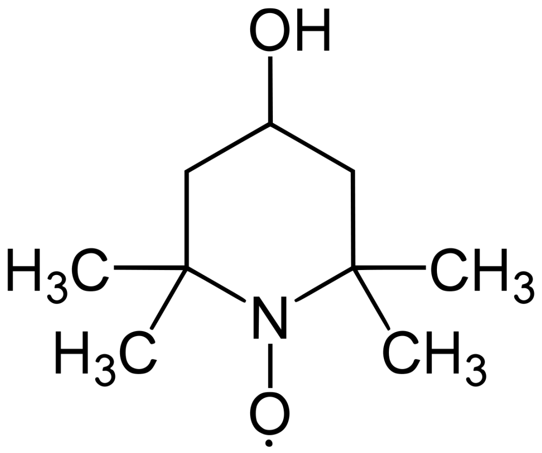

4-hydroxy-2,2,6,6-tetramethylpiperidine-N-oxyl

(Tempol)

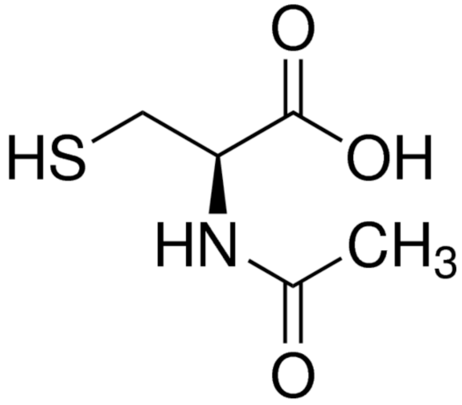

N-acetyl-L-cysteine

(L-NAC)

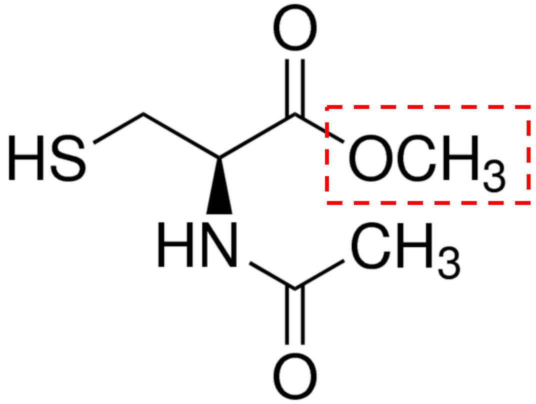

N-acetyl-L-cysteine methyl ester

(L-NACme)

Supplemental Figure 1. Structures of Tempol, N-acetyl-L-cysteine and N-acetyl-L-cysteine methyl ester.

Supplemental Figure 2

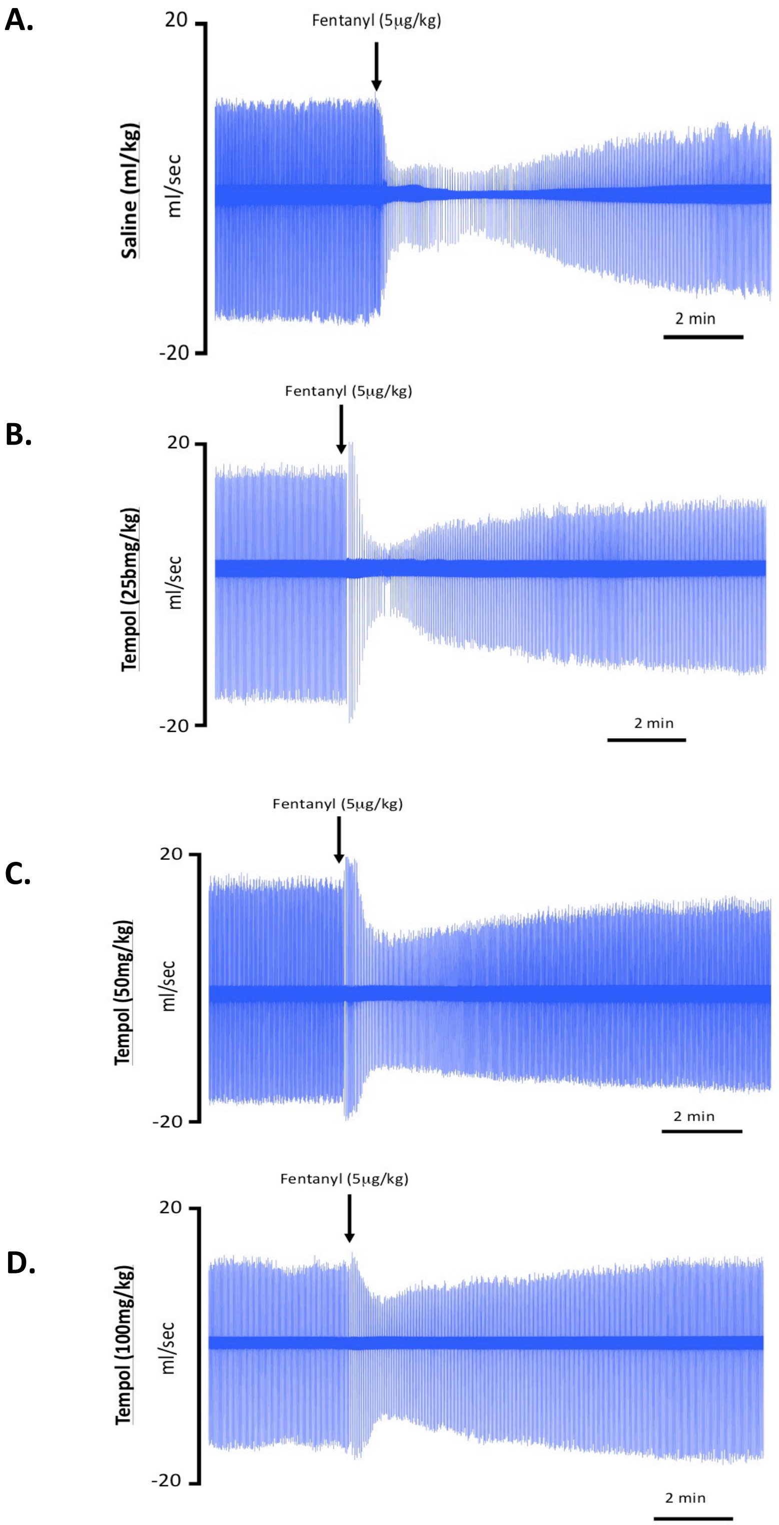

Supplemental Figure 2. Typical examples of the effects of an injection of fentanyl (5 µg/kg, IV) on ventilatory waveforms recorded from 4 separate rats that had received an IV injection of vehicle (saline, **Panel A**) or Tempol at 25 mg/kg (**Panel B**), 50 mg/kg (**Panel C**) or 100 mg/kg (**Panel D**).

Supplemental Figure 3

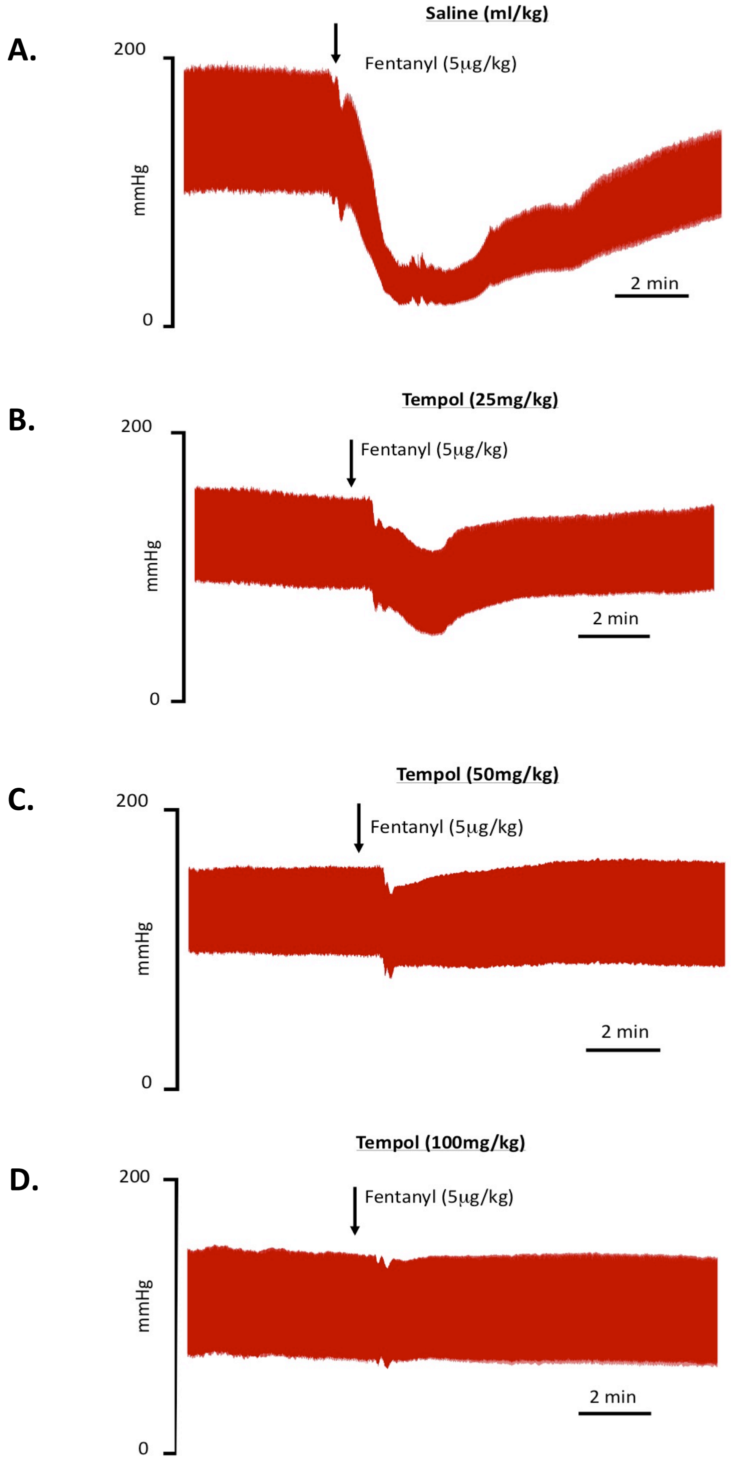

Supplemental Figure 3. Typical examples of the effects of an injection of fentanyl (5 μg/kg, IV) on arterial blood pressure waveform recorded from 4 separate rats that had received an IV injection of vehicle (saline, **Panel A**) or Tempol at 25 mg/kg (**Panel B**), 50 mg/kg (**Panel C**) or 100 mg/kg (**Panel D**).

Supplement Figure 4

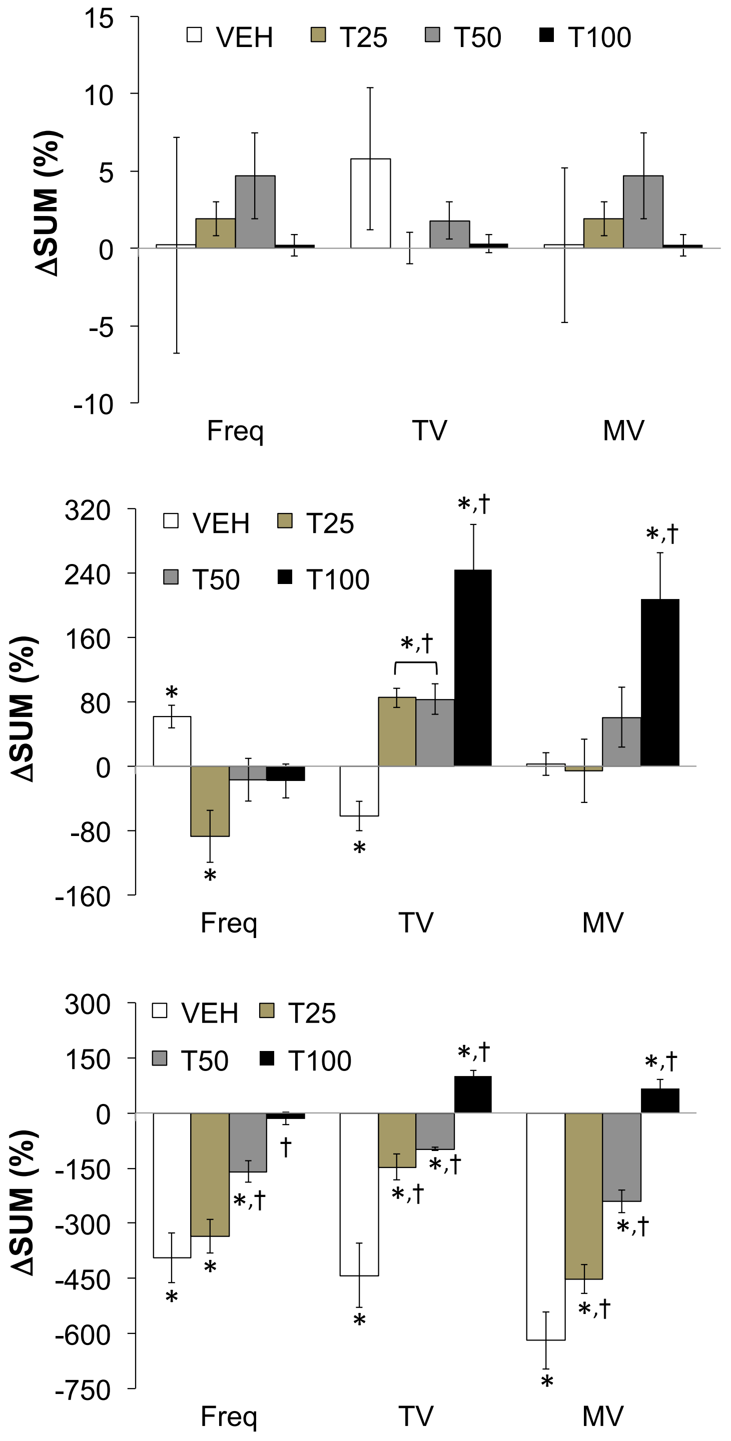

Supplemental Figure 4. A summary of the total changes in ventilatory parameters (the individual responses recorded at all time-points during each phase were summed together) elicited by bolus injections of vehicle (VEH) or Tempol (25, 50 or 100 mg/kg, IV; T25, T50, T100, respectively) and subsequent injection of fentanyl (5 μg/kg, IV) in isoflurane-anesthetized rats. There were 12 rats in the vehicle group, and 3, 5 and 12 rats in the 25, 50 and 100 mg/kg Tempol groups, respectively. Data are mean ± SEM. *P < 0.05, significant change. †P < T25, T50 and/or T100 versus vehicle.

Supplement Figure 5

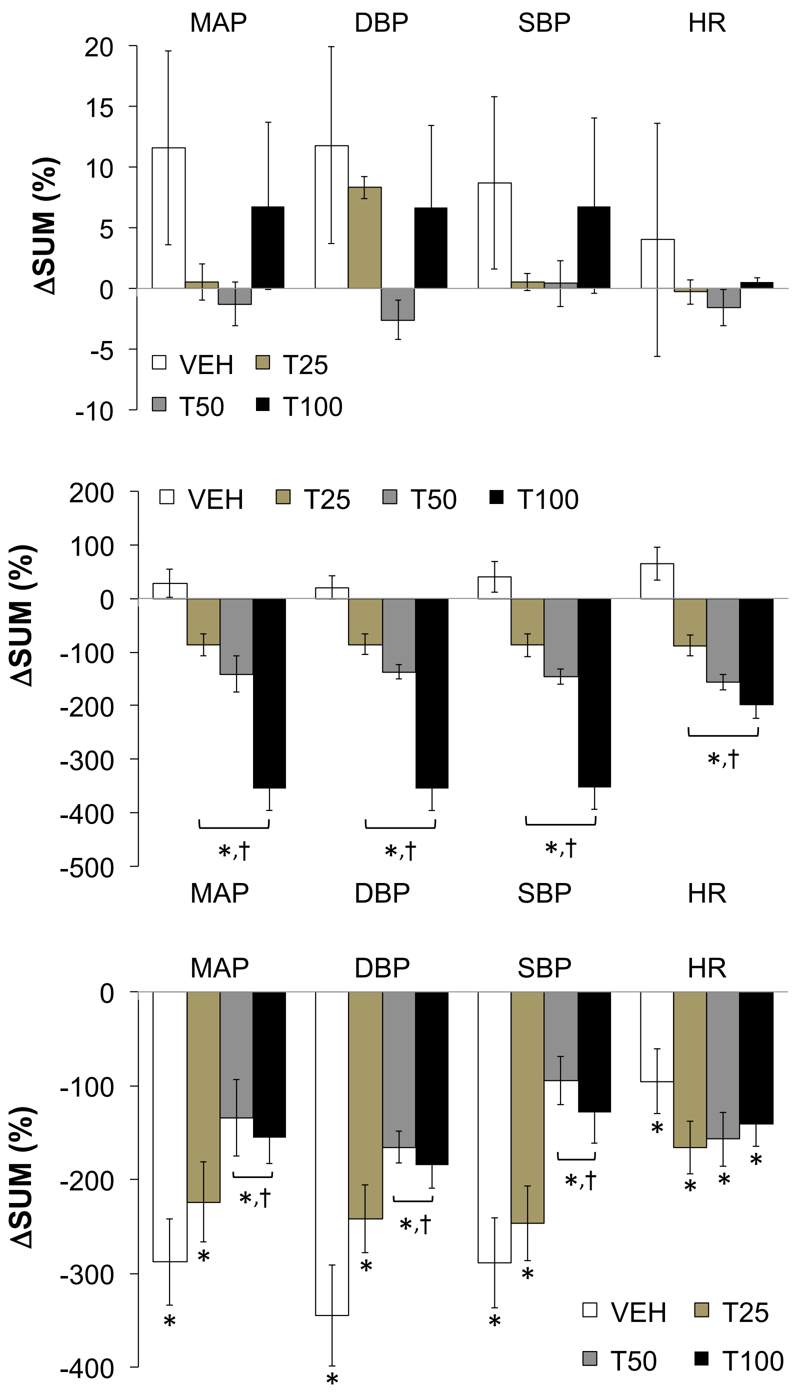

Supplemental Figure 5. A summary of the total changes (the individual responses recorded at all time-points during each phase were summed together) in mean (MAP), diastolic (DBP) and systolic (SBP) arterial blood pressures and heart rate elicited by bolus injections of vehicle (VEH) or Tempol (25, 50 or 100 mg/kg, IV; T25, T50, T100, respectively) and subsequent injection of fentanyl (5 μg/kg, IV) in isoflurane-anesthetized rats. There were 12 rats in the vehicle group, and 3, 5 and 12 rats in the 25, 50 and 100 mg/kg Tempol groups, respectively. The data are presented as mean ± SEM. *P < 0.05, significant change. †P < T25, T50 and/or T100 versus vehicle.

Supplement Figure 6

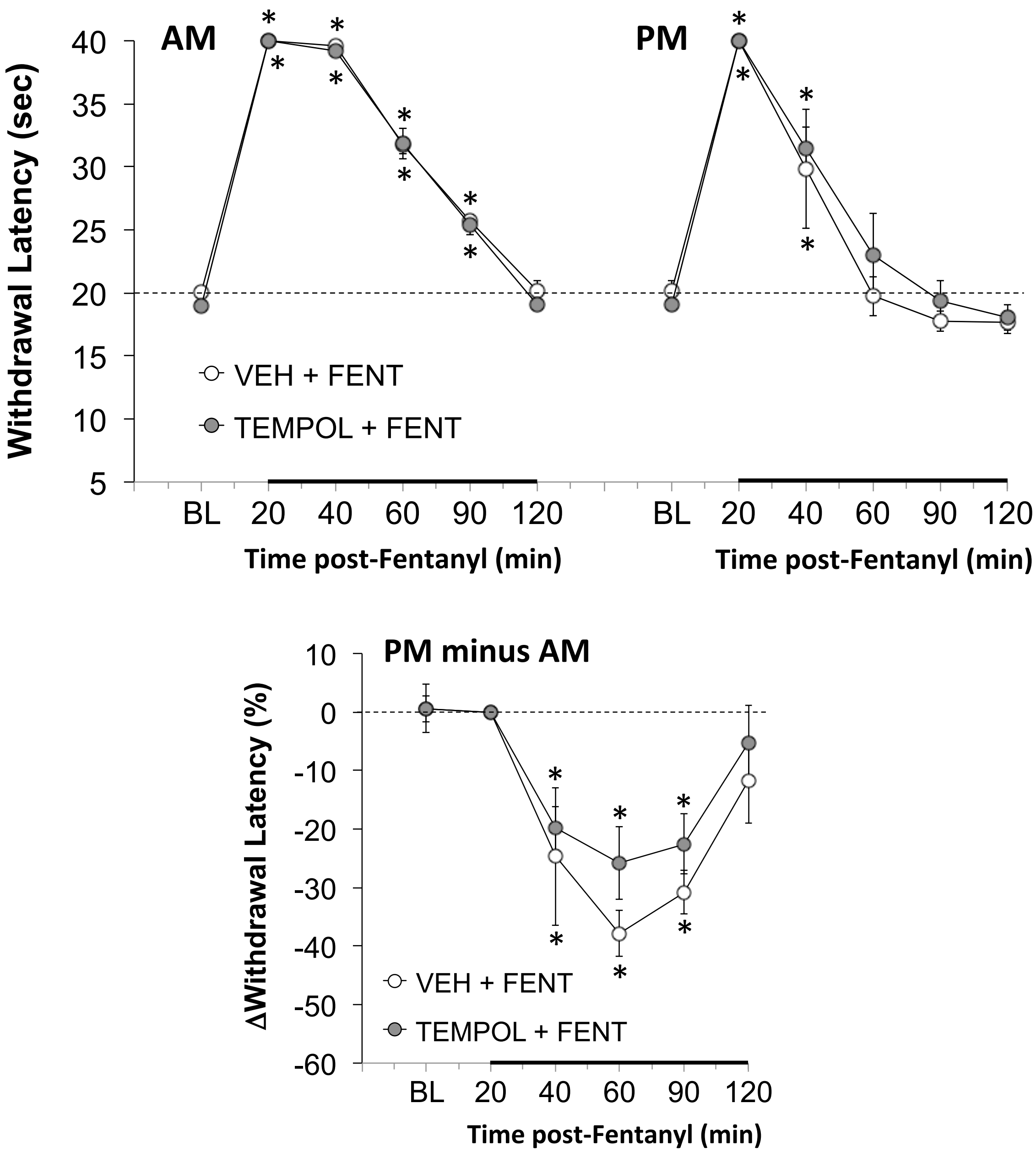

Supplemental Figure 6. A summary of the changes in PAW- withdrawal latency from baseline (BL) elicited by a bolus injection of fentanyl (25 μg/kg, IV) in conscious rats that had received an injection of Vehicle (VEH) or Tempol (100 mg/kg, IV) 20 minutes previously. The **top-left panel** shows the data from the first injection protocol commenced at 10 AM. The **top right panel** shows the data from a second injection of fentanyl in the same rats and 4 PM (no vehicle or Tempol pretreatment). The bottom panel shows arithmetic differences in paw-withdrawal latencies between the PM and AM studies. There were 6 rats in each group. The data are shown as mean ± SEM. *P < 0.05, significantly different from baseline. There were no differences between Vehicle- or Tempol-treated rats at any time point (P > 0.05, for all comparisons).

Supplement Figure 7

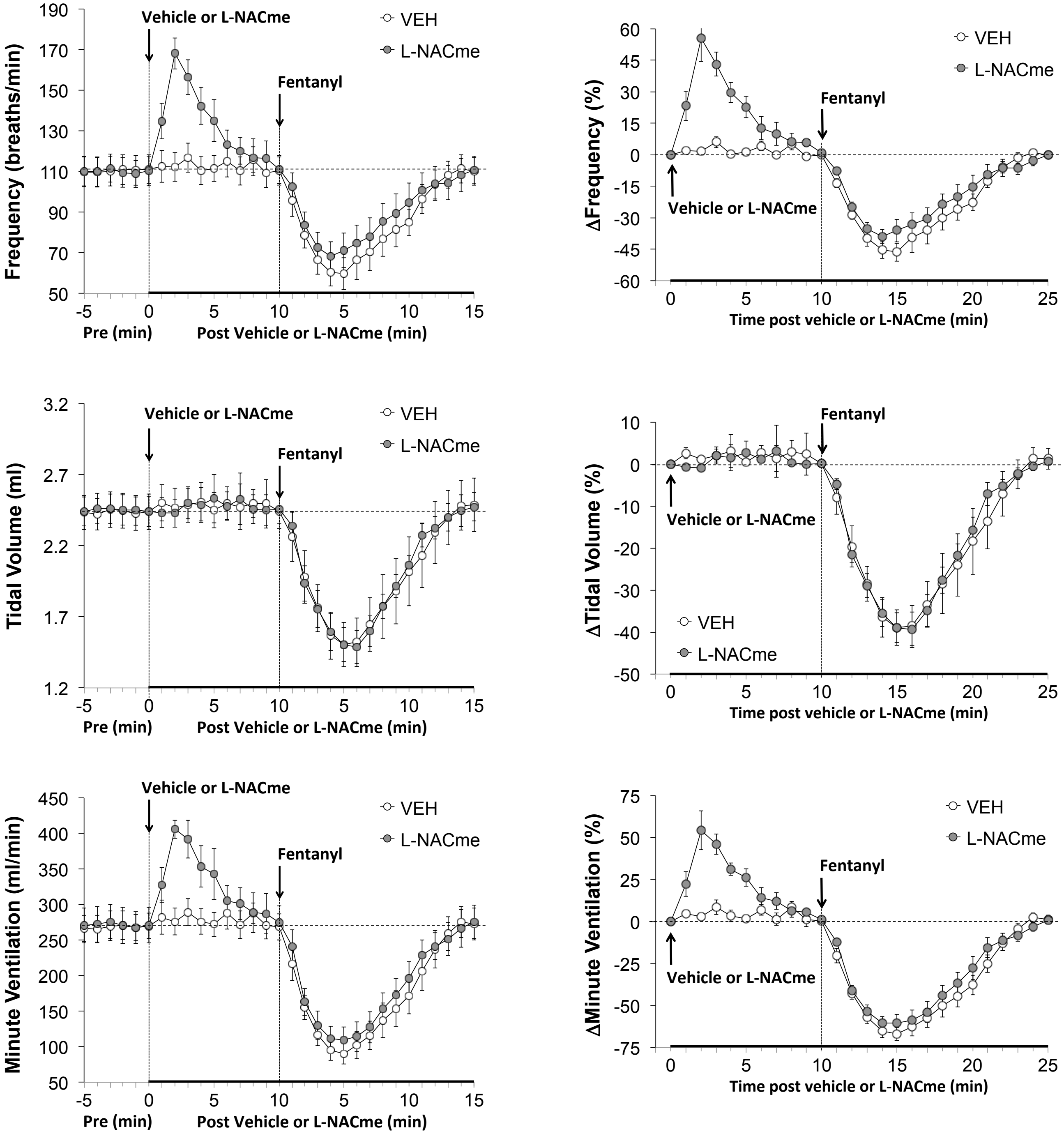

Supplemental Figure 7. A summary of the changes in frequency of breathing, tidal volume and minute ventilation elicited by bolus injections of vehicle or L-acetyl-N-cysteine (L-NACme, 500 μmol/kg, IV) and subsequent injections of fentanyl (25 μg/kg, IV) in freely-moving rats. There were 9 rats in each group. The data are presented as mean ± SEM. There were no differences between Vehicle- or Tempol-treated rats at any time point (P > 0.05, for all comparisons).

Supplement Figure 8

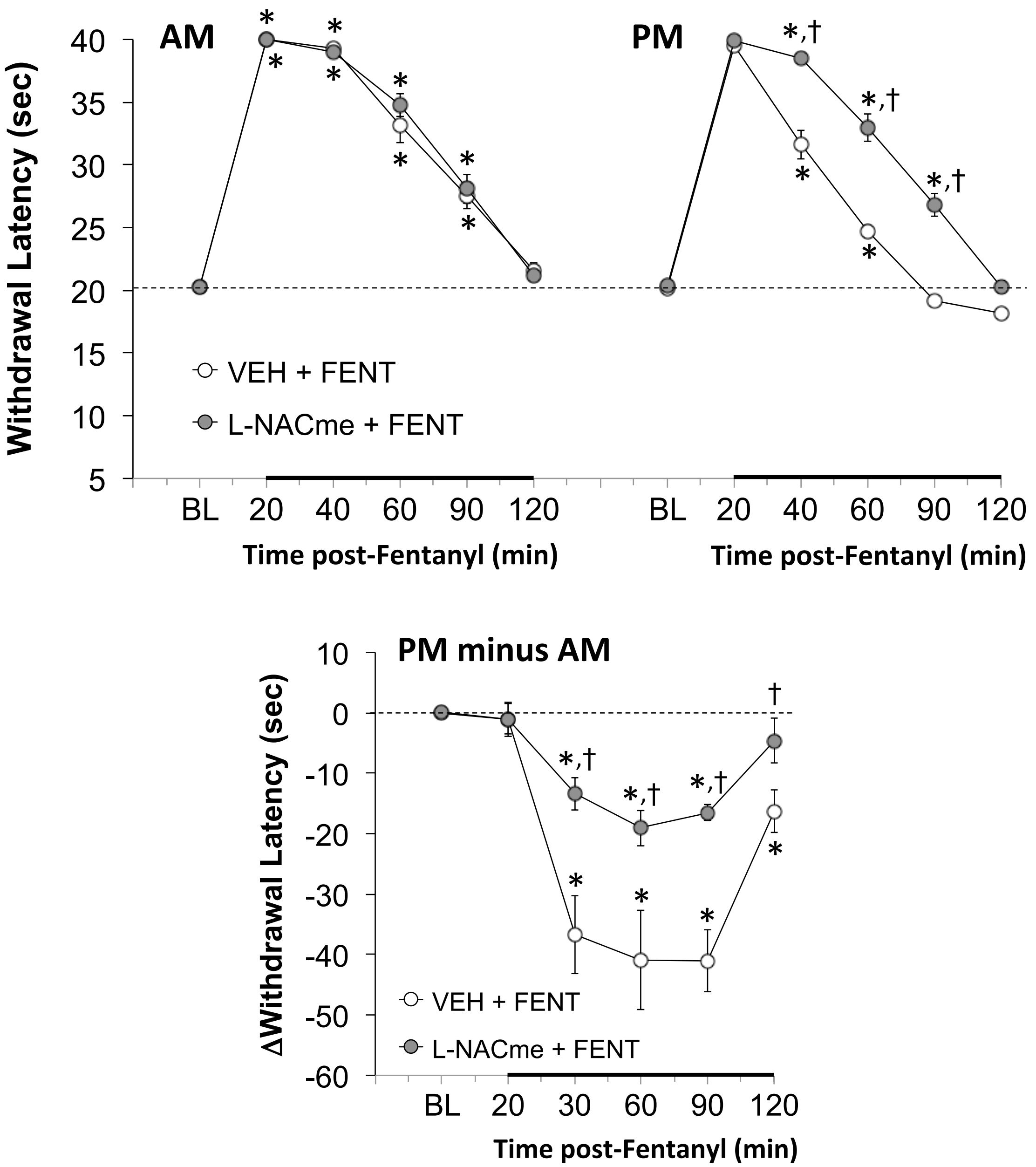

Supplemental Figure 8. A summary of the changes in paw-withdrawal latency from baseline (BL) elicited by a bolus injection of fentanyl (25 μg/kg, IV) in conscious rats that had received an injection of Vehicle or L-NACme (500 μmol/kg, IV) 20 minutes previously. The **top-left panel** shows the data from the first injection protocol commenced at 10 AM. The **top right panel** shows the data from a second injection of fentanyl in the same rats and 4 PM (no vehicle or L-NACme pretreatment). The bottom panel shows the arithmetic differences in paw-withdrawal latencies between the PM and AM studies. There were 6 rats in each group. The data are shown as mean ± SEM. *P < 0.05, significantly different from baseline. †P < L-NACme + fentanyl versus vehicle + fentanyl.

Supplemental Figure 9

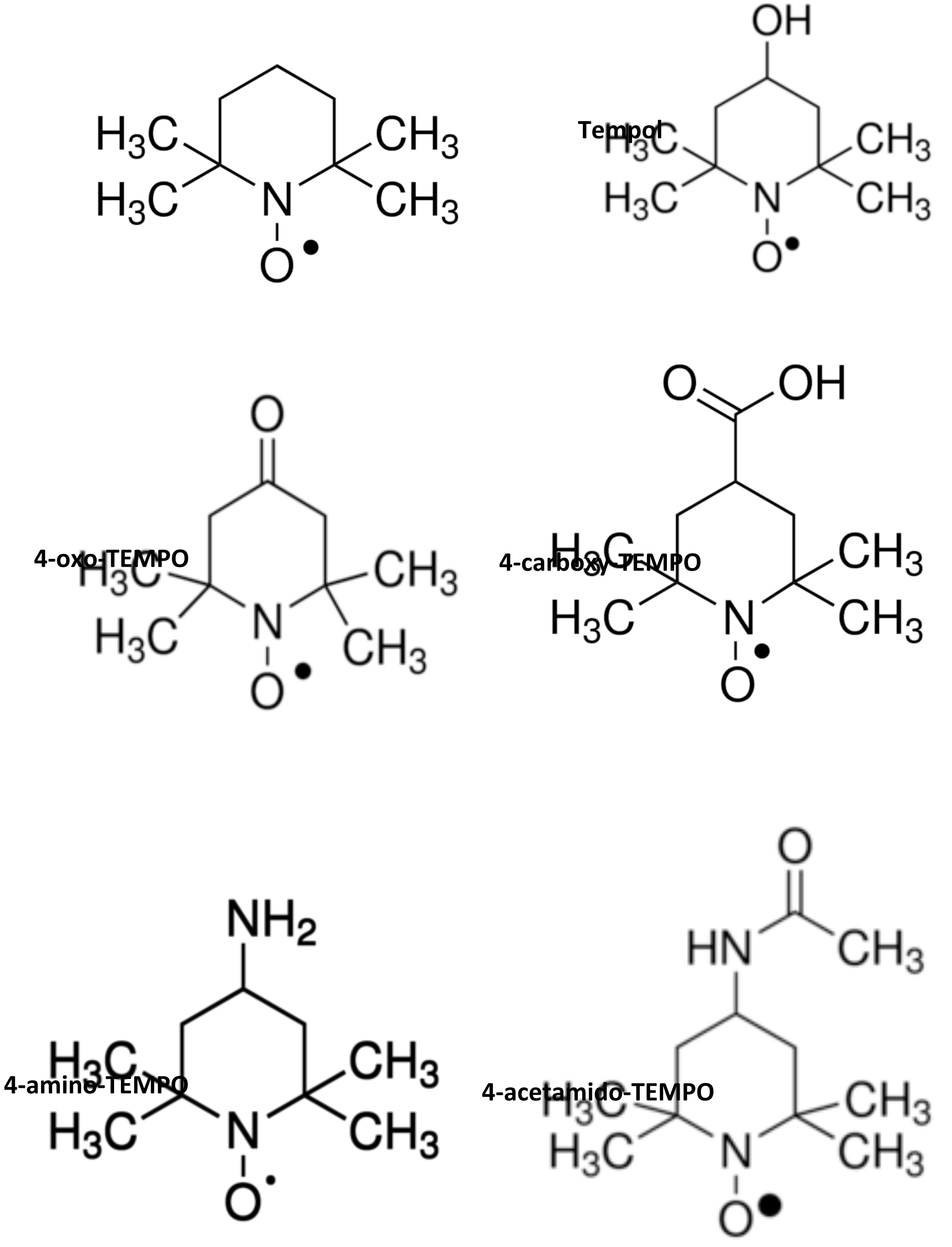

Supplemental Figure 9. Structures of Tempol and Tempol-related derivatives.
